# Associations Between Parent–Child Attachment and Psychosocial- and Health-Related Symptoms in Children with Functional Abdominal Pain Disorders

**DOI:** 10.3390/children12101371

**Published:** 2025-10-11

**Authors:** Camden E. Matherne, Tasha B. Murphy, Rona L. Levy, Shelby L. Langer, Joan M. Romano, Miranda A. L. van Tilburg

**Affiliations:** 1 Department of Psychiatry, University of North Carolina, Chapel Hill, NC 27514, USA; 2 School of Social Work, University of Washington, Seattle, WA 98105, USA; tbmurphy@uw.edu (T.B.M.); rlevy@uw.edu (R.L.L.); tilburg@med.unc.edu (M.A.L.v.T.); 3 Center for Health Promotion and Disease Prevention, Edson College of Nursing and Health Innovation, Arizona State University, Phoenix, AZ 85004, USA; shelby.langer@asu.edu; 4 Psychiatry and Behavioral Sciences, School of Medicine, University of Washington, Seattle, WA 98195, USA; jromano@uw.edu; 5 Cape Fear Valley School of Medicine, Methodist University, Fayetteville, NC 28311, USA; 6 Graduate Medical Education, Cape Fear Valley Health, Fayetteville, NC 28304, USA; 7 Division of Gastroenterology and Hepatology, University of North Carolina, Chapel Hill, NC 27514, USA

**Keywords:** functional abdominal pain, parental attachment, attachment-diathesis model, pediatric chronic pain

## Abstract

**Highlights:**

**What are the main findings?**

- In youth with Functional Abdominal Pain Disorders (FAPDs), less secure parental attachment is associated with more severe gastrointestinal symptoms, greater depressive symptoms, and more functional impairment.
- To varying degrees, pain catastrophizing mediated these relationships.

**What is the implication of the main finding?**

- Results support an attachment-diathesis model of pediatric FAPDs.
- Parent–child attachment and catastrophizing may be important treatment targets in children with FAPDs.

**Abstract:**

Background and Objectives: The attachment-diathesis model of chronic pain, which associates insecure attachment with pain catastrophizing and worse pain-related outcomes, is well-supported in adults. Although Functional Abdominal Pain Disorders (FAPDs) are common in youth, with symptoms influenced by the parent–child dynamic, an attachment-diathesis model of FAPDs is unexplored. The aim of this study was to investigate if insecure parental attachment is associated with pain catastrophizing and pain-related variables in youth with FAPDs. Methods: Baseline questionnaire data from an RCT of cognitive behavioral therapy for children with FAPDs (*n* = 200, 73% girls, 93% White, and a mean age of 11.2 years old) were used to examine relationships between parental attachment (subscales include Alienation, Trust, and Communication), catastrophizing, and pain-related variables (depression, disability, and gastrointestinal (GI) symptom severity). Results: Alienation was significantly correlated with depression (*r* = 0.39), GI symptom severity (*r* = 0.30), and disability (*r* = 0.22; *ps* < 0.05). Trust was also correlated with depression (*r* = −0.39), GI symptom severity (*r* = −0.19), and disability (*r* = −0.19; *ps* < 0.05). Communication was associated with depression (*r* = −0.30, *p* < 0.01). Catastrophizing mediated these associations, accounting for 22–89% of the relationship between attachment and pain-related variables. Conclusions: Children who report a less secure attachment to their parents report more physical and psychological symptomatology than children who describe their attachment as more secure. This association is partly explained by child catastrophizing. Results suggest that parent–child attachment and catastrophizing may be important treatment targets in children with FAPDs.

## 1. Introduction

Functional Abdominal Pain Disorders (FAPDs) involve centrally mediated chronic abdominal pain that is not fully explained by an underlying medical condition [1]. FAPDs affect approximately one in five children [2] and are the most common diagnosis in pediatric gastroenterology [3]. FAPDs are associated with increased disability, psychological distress, and reduced quality of life [4]. It is well-documented that social factors influence chronic pain, with parents in particular playing a unique role in influencing their child’s pain experiences and coping [5]. A review of the literature, including studies with FAPDs [4], indicates that pain-related parenting behaviors, such as attending to pain and pain catastrophizing (i.e., focusing on pain and magnifying the threat of pain), affect the child’s pain report, disability, and emotional response [6]. Thus, clarifying the mechanisms involved with these associations is important in the conceptualization and treatment of pediatric chronic pain.

The attachment-diathesis model [7] is an empirically based model of chronic pain [8] founded in attachment theory [9,10], which describes the child–parent bond as crucial to survival and development. In brief, attachment theory posits that the quality and consistency of how the caregiver responds to the child’s needs result in either a secure (i.e., caregiver consistently responsive) or insecure (i.e., caregiver inconsistently responsive) attachment style. Later in life, attachment schema (cognitions, emotions, and behaviors related to seeking safety or nurturance) can be activated by threats, eliciting approach or avoidance strategies depending on the quality of attachment. The attachment-diathesis model proposes that pain (the threat) triggers the attachment system, eliciting specific cognitive appraisals and behavioral responses to pain which, in turn, affect adjustment to pain. Accordingly, compared to securely attached individuals, those with insecure attachments are hypothesized to be more likely to appraise pain as a threat, as well as catastrophize (i.e., magnify the seriousness of the pain) and cope with pain by either avoiding social support or, conversely, overly relying on others for coping assistance. Such coping patterns may contribute to increased pain report, emotional distress, and disability.

Studies of adults with chronic pain provide support for the attachment-diathesis model. Insecure attachment styles are more common among chronic pain sufferers than healthy controls [11,12,13,14,15], including patients with irritable bowel syndrome, a common FAPD [15,16,17]. In these studies, insecure attachment was also associated with higher pain intensity [18,19,20], somatization [21], and worse pain-related perception such as pain catastrophizing and pain-related fear [19], as well as more severe clinical presentation, including greater co-morbid psychiatric symptoms, disability, poorer quality of life, and less adaptive pain coping [20,22,23,24,25]. In support of the attachment-diathesis model, in several studies, pain catastrophizing mediated the association between insecure attachment and pain-related outcomes [15,25,26], suggesting that pain appraisal is a critical component in the link between attachment style and chronic pain. See Cohen (2023) for a comprehensive review on the relationship between attachment styles and chronic pain in adults [27].

Only a few studies to date have examined associations between attachment and pain in pediatric samples, with findings generally mirroring the adult literature. Youth receiving inpatient treatment for chronic pain report less secure attachment compared to healthy controls [28]. Insecure attachment was also positively associated with pain severity among youth with migraine headache [29] and in a general community sample [30]. Consistent with the attachment-diathesis model, pain catastrophizing, as well as anxiety, mediated the relationship between attachment and pain [30].

Although FAPDs are common occurrences of pediatric chronic pain [31], attachment style in relation to adjustments in pediatric FAPDs is understudied. In a study of adolescents and adults with a history of childhood functional abdominal pain, current attachment anxiety was associated with poorer health-related quality of life, and this was mediated by pain threat and passive coping [32], consistent with the attachment-diathesis model. Only one study to date has examined attachment and pain outcomes in youth with FAPDs and found no difference in attachment style compared to healthy controls. However, the study included a small (*n* = 22) sample of youth with FAPDs that were also mixed with non-pain predominant functional gastrointestinal disorders, potentially confounding the results.

To address this knowledge gap, this study examined associations between attachment and pain in a large pediatric sample exclusively with FAPDs. In line with the attachment-diathesis model, we hypothesized that insecure attachment would be associated with poorer pain-related variables (increased pain severity, depression, and disability) and that these associations would be mediated by child pain catastrophizing.

## 2. Materials and Methods

### 2.1. Participants

Participants were children 7–17 years old with a physician diagnosis of abdominal pain of functional origin (defined as ≥3 episodes of recurrent abdominal pain during a 3-month period). Children had cohabited with their parent for the past 5 years or, in cases of divided custody, for at least half of the child’s lifetime. Participants were recruited from pediatric gastroenterology clinics to participate in a randomized controlled clinical trial of cognitive behavioral therapy (for trial details, see [33]). Children were excluded from participation if they did not speak English; had any chronic disease (e.g., Crohn’s, ulcerative colitis, pancreatitis, diabetes, epilepsy, and celiac sprue), physician-diagnosed lactose intolerance, or any positive physical or laboratory findings which would explain the abdominal pain; had any major surgery in the past year; or had a developmental disability impairing the ability to complete the study protocols. Parents provided consent for their children to participate, and children provided assent. IRB approval was obtained from all recruitment sites including Seattle Children’s Hospital, Seattle, WA, USA; Mary Bridge Children’s Hospital, Tacoma, WA, USA; and Goryeb Children’s Hospital, Morristown, NJ, USA.

### 2.2. Methods

Measures for the current study were collected at baseline prior to randomization. The first baseline questionnaire was completed on 4 December 2003, and the last follow-up questionnaire was completed on 8 June 2010. Children completed measures over the phone with a trained interviewer blinded to the study hypotheses, as has been previously implemented successfully [34,35]. To facilitate administration, families received a hard copy of the questionnaires for reference in advance of phone administration. In addition, questionnaires were also available to view on the study website. Caregivers reported on demographic and descriptive variables including illness duration based on the Questionnaire on Pediatric Gastrointestinal Symptoms (QPGS) [36]. Children reported on their own health-related and psychosocial functioning.

### 2.3. Child-Reported Measures

The People In My Life (PIML; [37]) is a child self-reported measure of attachment with their parents. It includes three subscales: Trust (10 items), Communication (5 items), and Alienation (5 items); for the purposes of this study, a 4-item version of the Alienation scale was administered, eliminating the item “I feel scared in my home.” The PIML does not identify categories of attachment (e.g., secure vs. insecure) but rather measures how much Trust/Communication (secure attachment) and Alienation (insecure attachment) the child reports. An exemplary item is “I trust my parents”, answered on a 4-point scale ranging from “Almost never or never true” to “Almost always or always true.”

The Catastrophizing subscale of the Pain Response Inventory (PRI; [38]) measured catastrophizing. This subscale consists of 5 items (e.g., “Think to yourself that it will never stop”) answered on a 0–4 scale ranging from “Never” to “Always.” Subscale items are averaged to obtain a mean subscale score (0–4).

The gastrointestinal symptom subscale of the Children’s Somatization Inventory (CSI; [39]) measured how much children were bothered by GI symptoms in the past 2 weeks. The gastrointestinal symptom subscale contains 7 items (nausea, constipation, diarrhea, abdominal pain, vomiting, bloating, and food making you sick) answered on a 0–4 scale (“not at all” to “a whole lot”). All items were summed to obtain a total score (0–28).

The 15-item Functional Disability Inventory (FDI; [40]) assessed how much physical health prevents children from functioning normally such as attending school. Items are answered on a 5-point scale from no trouble (0) to impossible (4). Total scores are computed by summing the item scores (0–60).

The Children’s Depression Inventory (CDI; [41]) is a widely used scale to measure pediatric depressive symptoms. It consists of 27 items answered on a 3-point scale (0–2). One item about suicidal ideation was omitted in the present study. Total scores were computed by summing the items (0–52).

### 2.4. Data Analysis

**Figure 1:**
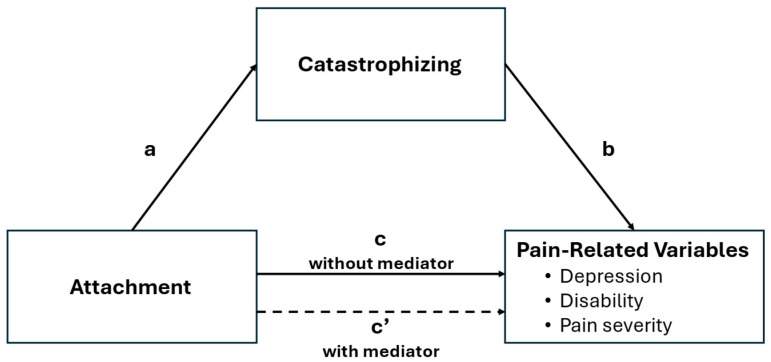
Mediation model.

Demographics were described with percentages and means (plus standard deviations). Means and standard deviations, as well as Spearman’s rank correlations between measures (due to non-normality of study variables), were calculated for attachment, GI symptom severity, depressive symptoms, disability, and catastrophizing. To test our hypotheses, mediation analyses were performed using Hayes’ PROCESS macro (http://www.processmacro.org/index.html, accessed on 1 June 2025). This is a regression-based path analytic analysis using boot strapping and Monte Carlo confidence intervals to estimate direct and indirect (mediation) effects. Separate mediation models (see Figure 1) were run for each of the three pain-related variables:

1. Attachment predicting depression (mediated by catastrophizing);
2. Attachment predicting GI symptom severity (mediated by catastrophizing);
3. Attachment predicting disability (mediated by catastrophizing).

Note that ‘prediction’ is used in a statistical sense and does not imply cause and effect, which cannot be determined from a cross-sectional study. All analyses were conducted in IBM SPSS 22.0.

## 3. Results

### 3.1. Sample Descriptives

Table 1 presents demographic characteristics and baseline means of all study variables. Based on parent report, the majority of child participants experienced pain in the upper (92%) or lower (89%) abdominal area for 4 or more months; a total of 65% and 69% experienced upper or lower abdominal pain, respectively, for 1 year or more. Bivariate correlations among study variables are shown in Table 2 and indicate that catastrophizing, depressive symptoms, GI symptom severity, and disability were all significantly correlated with one or more of the PIML subscales. As predicted, PIML Alienation (significant positive) and PIML Trust (significant negative) were associated with the three pain-related variables (depressive symptoms, GI symptom severity, and disability) as well as the hypothesized mediator (catastrophizing). Given that PIML Communication was only correlated significantly with depression, this subscale was excluded from the mediation analyses as a significant association is required to test mediation. Catastrophizing was significantly correlated with the pain-related variables as well.

### 3.2. Mediation Models

The results of the mediation analyses are depicted in Table 3 (PIML Alienation) and Table 4 (PIML Trust). In the tables and in Figure 1, the paths are defined as follows:

- Path a refers to the association between Attachment (PIML Alienation or Trust) and catastrophizing (hypothesized mediator).
- Path b refers to the association between catastrophizing and pain-related variables (depression, disability, or symptom severity).
- Path c refers to the association between attachment and pain-related variables (direct effect).
- Path ab indicates the indirect effect between attachment and pain-related variables (depression, disability, or symptom severity) through catastrophizing.
- Path c’ is the residual direct effect between attachment and pain-related variables after taking the effect of the mediator (catastrophizing) into account.

As can be seen in Table 3, in all three models, there was a significant indirect effect (ab) of Alienation on pain-related variables through catastrophizing (the mediator). Catastrophizing accounted for one-third (for symptom severity and depression) to more than half (for disability) of the effect of Alienation on pain-related variables. Similarly, there was a significant indirect effect of Trust on all three pain-related variables mediated through catastrophizing. Catastrophizing accounted for 22% of the effect in the model with depression, 56% in the model with symptom severity, and almost all the effect (89%) in the model with disability, suggesting that catastrophizing may be an especially important mediator for the association of PIML Trust with disability.

## 4. Discussion

This study tested hypotheses derived from the attachment-diathesis model in childhood FAPDs. Overall, findings supported our a priori hypotheses that attachment would be associated with pain outcomes and that these associations would be mediated by pain catastrophizing. Specifically, Alienation (insecure attachment) was associated with more severe GI symptoms and psychosocial pain variables (depressive symptoms and disability), while Trust (secure attachment) was associated with better outcomes on these same variables. These associations were mediated at least partially by pain catastrophizing.

Pain catastrophizing has been highly studied in relation to chronic pain [42]. The study findings replicated prior research evidencing associations between insecure attachment and pain catastrophizing in youth with chronic pain [30] and between pain catastrophizing and poor outcomes in youth with FAPDs [43,44,45]. Importantly, this is the first study to show (1) an association between insecure attachment and more severe pain-related symptoms in youth with FAPDs and (2) catastrophizing as a mediator in these associations, thus extending empirical support of the attachment-diathesis model [25,30] to youth with FAPDs.

There are several possible explanations for the relationships among attachment, catastrophizing, and pain symptoms in youth with chronic pain. Insecure attachment may increase the perceived threat value of pain and other symptoms, leading to higher levels of catastrophizing and greater dysfunction, as well as reduced self-efficacy for coping. Alternatively, behavioral expressions associated with catastrophizing, such as expressions of pain or distress, may function as a means of obtaining social support from significant others [46]. If children experience pain and perceive that parents are typically not responsive to their needs, they may feel a greater sense of threat and helplessness, leading them to express pain and distress overtly to obtain parental support. Indeed, we found that child-reported pain catastrophizing is greater in the context of more Alienation and less Trust (higher insecure attachment). However, it is also possible that chronic child expressions of distress and pain, especially in the absence of a specific diagnosis accounting for complaints, may lead to parental frustration, resentment, and a reduction in support. Longitudinal studies are needed to clarify how these relationships may interact over time to influence negative pain-related outcomes.

The relative effect sizes in the current study suggest that pain catastrophizing is a particularly important mediator for the relationship of attachment to disability. Indeed, the association between pain catastrophizing and disability has been well-documented [42]. Children who report similar levels of pain intensity can vary widely in the amount of pain-related disability, such as school absence [47]. For this reason, in treatment for FAPDs, pain catastrophizing and the return to normal activities are viewed as important treatment targets [48]. Behavioral treatments to date have largely focused on cognitive behavioral therapy (CBT), which improves patient pain- and psychosocial-related outcomes from pre- to post-intervention [49], yet a substantial portion of patients remain symptomatic or relapse, underscoring the importance of developing additional treatment options. Our findings suggest that addressing parent–child interactions around pain and strengthening parent–child trust and attachment may be useful additional treatment targets in FAPD behavioral interventions. Future studies are needed to assess if non-CBT interventions targeting attachment, such as interpersonal psychotherapy, a treatment founded in attachment theory and effective in reducing anxiety and depressive symptoms [50], may potentiate the effects of CBT or serve as an alternative approach for CBT non-responders.

There are several limitations to this study: First, the sample included youth and parents seeking treatment in an intervention trial, who may reflect a more symptomatic or distressed population than those not seeking treatment and, hence, may not be representative of the larger FAPD population. However, scores on symptom severity and disability were fairly low in our sample on average. Also, the study sample was primarily female and Caucasian, and thus the results may not generalize to more diverse populations. Second, this study was cross-sectional in nature and therefore cannot determine cause and effect. Longitudinal studies are needed to test whether insecure attachment leads to poorer outcomes or if greater levels of symptomatology contribute to disruption in parent–child attachment as well as to catastrophizing. Third, we did not examine any impact of parent–child-specific attachment (i.e., mother vs. father and daughter vs. son) on pain-related symptoms. This is a limitation given past research indicating sex-specific attachment relationships with mental health outcomes [51,52]. Lastly, there are limitations related to study measures. All study measures were self-reported and thus may show some degree of association due to shared method variance. The findings are further limited by the use of telephone-based assessment administration, an increasingly popular, reliable, less burdensome, and cost-effective assessment option used by our group and others [39,53,54,55,56], although it is not validated for all study measures. In addition, although we used validated scales commonly used in youth as young as 7 years old (e.g., [34,54,57,58]), the measures have not been validated for use in children below 8 years old. Lastly, as managing child safety concerns was beyond the scope of the study protocol, the PIML Alienation scale did not include the item assessing safety at home, and the CDI did not include the item assessing suicidality, thus differing from the original validated scales. Future studies would benefit from the addition of measures from other sources, such as parents, or objective measures of behavior.

## 5. Conclusions

In conclusion, we found associations between child–parent attachment and pain-related variables in children with FAPDs, and that catastrophizing played a significant mediational role in many of these associations. This study adds to our existing knowledge of the biopsychosocial model of FAPDs [1] by indicating that the quality of the parent–child relationship as well as child catastrophizing influences symptom presentation. Future studies are needed to clarify how these factors interact over time to determine the differential effects of empirically relevant factors, such as parent/child sex and age, as well as FAPD diagnostic categories (i.e., irritable bowel syndrome vs. abdominal migraine), and the potential impact of attachment on the transition from acute to chronic pain. Ultimately, future studies are needed to determine how attachment-focused interventions may be harnessed to improve outcomes in children with chronic abdominal pain.

## Figures and Tables

**Table 1:** Sample demographics (*n* = 155).

Study Variable	% or Mean (SD)
Age	11.20 (2.6)
Sex (% female)	73%
Race (% White)	93%
Ethnicity (% Hispanic)	5%
PIML	
Trust	3.59 (0.4)
Communication	3.26 (0.6)
Alienation	1.79 (0.6)
Symptom severity (CSI)	8.3 (4.8)
Catastrophizing (PRI)	1.59 (0.9)
Depression (CDI)	9.78 (6.5)
Disability (FDI)	11.32 (9.0)

Note: PIML = People In My Life, CSI = Child Symptom Inventory, PRI = Pain Response Inventory, CDI = Children’s Depression Inventory, FDI = Functional Disability Index.

**Table 2:** Correlations among study variables.

	1	2	3	4	5	6	7
1. PIML Communication	1						
2. PIML Alienation	−0.48 **	1					
3. PIML Trust	0.78 **	−0.55 **	1				
4. Catastrophizing (PRI)	−0.12	0.25 **	−0.17 *	1			
5. Depression (CDI)	−0.30 **	0.39 **	−0.39 **	0.46 **	1		
6. Symptom severity (CSI)	−0.07	0.30 **	−0.19 **	0.43 **	0.45 **	1	
7. Disability (FDI)	−0.13	0.22 **	−0.19 **	0.38 **	0.41 **	0.55 **	1

Note: PIML = People In My Life, CSI = Children’s Somatization Inventory, PRI = Pain Response Inventory, CDI = Children’s Depression Inventory, FDI = Functional Disability Inventory, * *p* < 0.05, ** *p* < 0.01.

**Table 3:** Results of mediation analyses using PIML Alienation as the predictor and catastrophizing as the mediator.

	Path C	Path A	Path B	(A × B)	C’	
Child-reported outcome	Effect of predictor on pain-related variable	Effect of predictor on mediator	Effect of mediator on pain-related variable	Indirect effect; 95% CI	Direct effect	Ratio of indirect effect to total effect
GI symptom severity (CSI)	2.81 (0.53) ***	0.43 (0.10) ***	2.21 (0.36) ***	0.96 (0.29); 0.43,1.56	1.85 (0.51) ***	0.34
Disability (FDI)	3.17 (1.02) **	0.43 (0.10) ***	4.07 (0.71) ***	1.77 (0.60); 0.73, 3.02	1.40 (1.00) ^NS^	0.56
Depression (CDI)	4.47 (0.69) ***	0.43 (0.10) ***	3.23 (0.46) ***	1.40 (0.42); 0.64, 2.26	3.06 (0.64) ***	0.31

Note: Results presented as estimate (standard error); CSI = Children’s Somatization Inventory, FDI = Functional Disability Inventory, CDI = Children’s Depression Inventory, ** *p* < 0.01, *** *p* < 0.001, ^NS^ *p* ≥ 0.05.

**Table 4:** Results of mediation analyses using PIML Trust as the predictor and catastrophizing as the mediator.

	Path C	Path A	Path B	(A × B)	C’	
Child-reported outcome	Effect of predictor on pain-related variable	Effect of predictor on mediator	Effect of mediator on pain-related variable	Indirect effect; 95% CI	Direct effect	Ratio of indirect effect to total effect
GI symptom severity (CSI)	−1.58 (0.78) *	−0.35 (0.14) *	2.56 (0.36) ***	−0.88 (0.35); −1.61, −0.23	−0.69 (0.71) ^NS^	0.56
Disability (FDI)	−1.70 (1.47) ^NS^	−0.35 (0.14) *	4.37 (0.69) ***	−1.51 (0.63); −2.86, −0.36	−0.19 (1.36) ^NS^	0.89
Depression (CDI)	−5.50 (0.98) ***	−0.35 (0.14) *	3.52 (0.44) ***	−1.22 (0.49); −2.23, −0.31	−4.28 (0.87) ***	0.22

Note: Results presented as estimate (standard error); CSI = Children’s Somatization Inventory, FDI = Functional Disability Inventory, CDI = Children’s Depression Inventory * *p* < 0.05, *** *p* < 0.001, ^NS^ *p* ≥ 0.05.

## Data Availability

The data supporting the conclusions of this article will be made available by the authors upon request.

## Abbreviations

The following abbreviations are used in this manuscript:

FAPDs	Functional abdominal pain disorders
PIML	People In My Life
PRI	Pain Response Inventory
CSI	Children’s Somatization Inventory
FDI	Functional Disability Inventory
CDI	Children’s Depression Inventory
